# The Annual Meeting of the American Medical Association

**Published:** 1886-04

**Authors:** 


### EDITORIAL.

### THE ANNUAL MEETING OF THE AMERICAN MEDICAL ASSOCIATION.

The approaching meeting of the American Medical Association promises to be of unusual interest and importance. The Committee on Organization of the International Medical Congress, appointed by the Association, having performed its duty, is prepared to report to the Association the fact that the congress has been organized, and that the affairs of the congress are now in the hands of the Executive Committee, consisting of the general officers of the congress and the presidents of the different sections. The Association can then accept the report and discharge its committee. After that the Association can with propriety declare its official connection with the congress to be terminated, having discharged the duty which it was requested to undertake, in inviting the Congress and providing for its organization. By such action the Association can avoid any question of financial responsibility in connection with the congress if there should, unfortunately, prove to be a deficiency of funds subscribed for the expenses attending it. Such a course of action on the part of the Association will, no doubt, remove all occasion for further wrangling over the congress, and materially add to the scientific value of the meeting. It is stated that the papers to be read in the various sections are already numerous and evince an unusual degree of original research. This state of affairs is probably due to the judicious selection of the chairmen and secretaries of the various sections. Medical politics played but an unimportant rôle in their election at New Orleans. To further conserve the interests of the profession, an amendment to the by-laws was offered by Dr. Foster Pratt, of Michigan, two years ago. The substance of the amendment is that each section shall nominate its Chairman and its Secretary—all other nominations to be made, as now, by the nominating committee. The actual workers in the sections are in a better position to determine the qualifications of their own presiding and recording officers than the nominating committee is.

All who were present at the New Orleans meeting last year, will remember with pleasure the scholarly address of the retiring PRESIDENT of the ASSOCIATION, DOCTOR CAMPBELL, of Georgia. One of the suggestions contained in this address referred to the creation of a new section, to be known as the Section on Medical Jurisprudence. Dr. I. N. Quimby, of New Jersey, embodied Dr. Campbell's suggestion in a proposed amendment to the by-laws. Every American medical practitioner is so thoroughly convinced, either through his own experience or that of his professional friends, of the vital importance of the subject, that Dr. Quimby's proposed amendment requires no discussion.

A letter over the signature "Branch", appeared in the columns of the *Journal of the American Medical Association*, February 6th, 1886, which invited attention to the desirability of making material changes in the plan of organization of the Association. It was proposed in this letter to adopt the plan of the British Medical Association, and divide our national society into a number of branches. Able letters upon the topic, by Dr. James H. Parkinson, of Sacramento, Dr. John B.

Hamilton, of Washington, Dr. J. M. Keller, of Hot Springs, and others, have appeared in subsequent issues of the *Journal*. The discussion is thus fairly inaugurated, and promises to be at least instructive. The editor of the *Journal*, in a timely and judicious editorial, appearing February 27th, 1886, mentions the fact that the same plan engaged the attention of the committee appointed by the National Medical Convention in 1846. After careful consideration of the subject, the committee reported the plan of organization, which, with some modifications, has endured to the present time. Now, without attempting to debate the question at the present time, we are under the impression that "an efficient and general organization of the profession" can "be founded on a proper application of the principle of *representation* from local organizations to state and from the state to the national as the union of the whole,"—the essential principle of the American Medical Association, as at present organized. We believe that this principle of organization is better adapted to the wants of the American Medical Association than a national organization, with a council self-constituted and capable of self-perpetuation, and a broader nominal membership,—the essential principle on which the British Medical Association is at present based. The subject is before the profession, and it is probable that it will command attention in the next meeting of the Association, which opens on the fourth day of May.

